# Containing the crisis: mentalisation-based therapy reduces acute service use for emotionally unstable personality disorder in Northern Ireland

**DOI:** 10.1192/bjb.2025.10191

**Published:** 2026-02

**Authors:** Adam Flynn, Cedar Andress, Owen McNeill

**Affiliations:** Northern Ireland Medical & Dental Training Agency, Belfast, UK.; Northern Ireland Medical & Dental Training Agency, Belfast, UK; Northern Health and Social Care Trust, Antrim, UK

Emotionally unstable personality disorder (EUPD) represents a significant clinical and service-level challenge in Northern Ireland, where the prevalence of mental health disorders is estimated to be 25% higher than in England.^1^ In line with Northern Ireland strategy, the Northern Health and Social Care Trust implemented mentalisation-based therapy (MBT) in 2012 as an evidence-based intervention, rooted in attachment theory, to address these complex needs.^2^ However, robust local evidence of its real-world impact has remained limited, hindering effective regional service planning under the 10-year Mental Health Strategy 2021–2031. We conducted a single-site pre–post service evaluation to assess the impact of an 18-month MBT programme on acute service utilisation in individuals with EUPD.

## Methods and key findings

We retrospectively evaluated data for 19 patients who had completed the full MBT programme between 2013 and 2018. We compared acute service use (in-patient admissions, crisis or emergency department contacts, and same-day assessments in the 18 months pre-therapy versus the 12 months post-therapy completion. Pre-therapy data were normalised to 12 months for direct comparison. Statistical significance was determined using a one-tailed Wilcoxon signed rank test (*P* ≤ 0.05). Data were analysed using IBM SPSS Statistics software version 31 available at https://www.ibm.com/products/spss-statistics.

The evaluation demonstrated a significant reduction in acute service use post-MBT, consistent with systematic reviews on psychotherapies for borderline personality disorder:^3^

- In-patient admissions: days per patient decreased from a normalised mean of 14.49 days pre-therapy to 3.68 days post-therapy. This statistically significant reduction of 74.6% (*P* = 0.005) indicates substantial clinical stability and a major reduction in resource burden.
- Crisis/emergency department contacts: mean crisis resolution/home treatment and emergency department contacts declined from a normalised mean of 1.76 pre-therapy to 0.58 post-therapy. This statistically significant reduction of 67% (*P* = 0.009) aligned closely with outcomes reported by other naturalistic MBT studies in Ireland.^4^

The reduction in same-day assessments was modest and not statistically significant (*P* = 0.458), although this finding was potentially limited by data fragmentation and a small sample size (*n* = 8) for this specific metric. Selection bias was also a key limitation, as the cohort only included programme completers.

The authors assert that all procedures contributing to this work comply with the ethical standards of the relevant national and institutional committee on human experimentation and with the Helsinki Declaration of 1975, as revised in 2008. The authors assert that ethical approval for publication of this service evaluation has been sought and deemed not necessary for publication by their local Ethics Committee Research Governance Manager at the Northern Health and Social Care Trust on 21 July 2025. All patient information was anonymised, and specific consent was not required.

## Conclusion and policy implications

This service evaluation provides crucial local evidence that MBT, as delivered within public services in Northern Ireland, effectively reduces crisis-driven behaviours and acute service reliance in individuals with EUPD. The findings are consistent with those of randomised controlled trials comparing MBT with structured case management in mainstream UK settings.^5^

Despite this success, access to specialist therapies such as MBT remains a postcode lottery across Northern Ireland’s health and social care trusts, with significant variability in staffing and implementation. This disparity undermines the goals of the Northern Ireland Mental Health Strategy 2021–2031, which advocates for integrated, scalable and evidence-based psychological treatments. Sustained investment is critical to maintain therapeutic quality, standardise training (mirroring UK-wide curriculum efforts) and ensure equitable access across all trusts. Our data support the crucial role of MBT as a scalable, evidence-based intervention for complex emotional and relational needs in the region.
